# 伴SH2B3基因纯合胚系突变的骨髓增殖性肿瘤1例报告并文献复习

**DOI:** 10.3760/cma.j.cn121090-20251019-00472

**Published:** 2026-04

**Authors:** 伟 张, 蓉 李, 娟 田, 丽君 蒋, 洁 白, 义海 顾, 园园 王, 燕 苏, 叶琼 李, 毅苗 薛, 俊敏 陈, 玉萍 魏

**Affiliations:** 1 宁夏回族自治区人民医院（宁夏医科大学附属自治区人民医院）血液内科，银川 750021 Department of Hematology, People's Hospital of Ningxia Hui Autonomous Region, Ningxia Medical University, Yinchuan 750021, China; 2 宁夏回族自治区人民医院（宁夏医科大学附属自治区人民医院）宁夏老年病中心，银川 750021 Ningxia Geriatric Center, People's Hospital of Ningxia Hui Autonomous Region, Ningxia Medical University, Yinchuan 750021, China; 3 宁夏回族自治区人民医院（宁夏医科大学附属自治区人民医院）骨髓病理室，银川 750021 Bone Marrow Pathology Room, People's Hospital of Ningxia Hui Autonomous Region, Ningxia Medical University, Yinchuan 750021, China

## Abstract

回顾性分析了国内首例由SH2B3基因纯合胚系突变驱动的骨髓增殖性肿瘤（MPN）患者的临床资料，旨在加深对该罕见MPN亚型的认识。患者为59岁女性，因间断乏力伴发热3年加重4 d入院。二代测序结果显示其存在SH2B3基因c.1481C>A p.S494*纯合胚系突变［变异等位基因突变频率（VAF）100％］，并通过家系分析证实为胚系来源，其子女均为SH2B3基因杂合突变携带者，表型正常。结合血液学检查及骨髓病理，明确诊断为MPN，临床主要表现为发热、盗汗、WBC及PLT增高、脾大。SH2B3基因作为JAK2的负调控因子，其失活突变导致SH2B3蛋白对JAK2/STAT信号通路的负调控作用减弱，从而引起该信号通路的持续激活，诱导MPN的发生。予JAK1/JAK2抑制剂芦可替尼20 mg每日2次治疗后，患者体温恢复正常，乏力缓解，盗汗消失，脾脏减小，治疗效果显著。

## Abstract (en)

This study retrospectively analyzed the clinical data of the first reported case in China of myeloproliferative neoplasm（MPN）driven by a homozygous germline SH2B3 mutation, aiming to deepen the understanding of this rare MPN subtype. The patient was a 59-year-old female admitted due to intermittent fatigue and fever for 3 years, which had worsened 4 days prior to admission. Next-generation sequencing revealed a homozygous germline SH2B3 gene mutation（c.1481C>A, p.S494*）with a variant allele frequency（VAF）of 100％. Family analysis confirmed its germline origin, and her children were all heterozygous carriers of the SH2B3 mutation with normal phenotypes. Based on hematological examination and bone marrow pathology, MPN was definitively diagnosed. The main clinical manifestations included fever, night sweats, elevated WBC and PLT, and splenomegaly. As a negative regulator of JAK2, inactivating mutations in the SH2B3 gene lead to weakened negative regulation of the JAK2/STAT signaling pathway by the SH2B3 protein, thereby causing continuous activation of this pathway and inducing the development of MPN. After treatment with the JAK1/JAK2 inhibitor ruxolitinib 20 mg twice daily, the patient's body temperature returned to normal, fatigue and night sweats resolved, and spleen size decreased, demonstrating significant therapeutic efficacy.

骨髓增殖性肿瘤（MPN）是以分化相对成熟的一系或多系骨髓细胞异常增殖为特点的一类造血干细胞克隆性疾病。已证实97％～99％的真性红细胞增多症（PV）患者存在JAK2V617F或JAK2 12-16外显子突变，80％～90％的原发性血小板增多症（ET）和原发性骨髓纤维化（PMF）患者存在JAK2V617F、MPL、CALR基因突变，这些基因通过激活JAK/STAT信号通路，促进骨髓细胞增殖[1]。SH2B3基因（又称LNK基因）是JAK2的负调控因子，失活突变会导致SH2B3蛋白对JAK2/STAT信号通路的负调控作用减弱，从而导致该信号通路的持续激活，诱导MPN的发生[2]。近期我科诊治1例SH2B3基因纯合胚系突变导致的MPN患者，系国内首次发现，现对其基因突变特征及致病的分子机制、家系、临床资料等分析如下。

## 病例资料

患者，女性，59岁，主因“间断乏力伴发热3年加重4 d”于2022年10月24日入院。患者2019年7月无明显诱因出现乏力、盗汗、发热，当时未予重视。2020年8月病情加重，就诊外院，查腹部B超示：脾大，门静脉增宽，余未见异常；胸部CT示肺间质性改变，给予抗感染治疗效果欠佳。随后就诊我院呼吸内科，查血常规、肝肾功能、电解质、免疫球蛋白、补体均正常；EB病毒DNA、结核菌素试验、布氏杆菌凝集试验、自身抗体谱、肌炎抗体谱、Coombs试验均阴性，抗核抗体阳性（胞浆颗粒型1∶1 000），诊断：间质性肺炎和结缔组织病，考虑发热与自身免疫性疾病相关，给予抗感染联合甲泼尼龙治疗后好转出院。院外仍间断乏力、盗汗。

2022年10月20日患者再次发热，最高39.0 °C，乏力盗汗明显，即就诊我院。门诊查血常规示：WBC 8.21×10^9^/L，中性粒细胞6.45×10^9^/L，HGB 136 g/L，PLT 547×10^9^/L；腹部B超示：脾大（厚5.0 cm，上下径19.8 cm），遂以“脾大原因待查”收住入院。查体：体温38.3 °C，心率90次/min，呼吸19次/min，血压124/84 mmHg（1 mmHg = 0.133 kPa），神清，全身皮肤黏膜正常，周身浅表淋巴结未触及肿大，心肺查体未见异常，腹部平坦，无压痛及反跳痛，肝肋缘下未触及，脾脏肋缘下可触及肿大（Ⅰ线14.5 cm，Ⅱ线16.5 cm，Ⅲ线−1.5 cm），双下肢无水肿。末梢血涂片示：中性杆状核粒细胞12.0％，中性分叶核粒细胞77.0％，淋巴细胞5.0％；血生化示：β_2_微球蛋白8.33 mg/L，乳酸脱氢酶344 U/L；血清铁蛋白185 µg/L；超敏C反应蛋白36.24 mg/L，降钙素原0.03 ng/L；抗核抗体阳性；血清免疫固定电泳阴性。查肝肾功能、电解质、免疫球蛋白正常；抗链球菌溶血素O、类风湿因子均阴性；凝血全套、肌钙蛋白T、N末端B型利钠肽前体均正常；乙肝DNA、抗丙肝抗体阴性；尿常规正常。骨髓涂片：有核细胞增生Ⅲ级，粒系占71％，红系占16％，粒红比为4.4∶1，粒系增生明显活跃，各阶段细胞均可见，早幼粒细胞比例偏高（9％），以中性杆状核及分叶核粒细胞为主；红系增生活跃，以中晚幼红细胞为主，成熟红细胞形态大致正常，淋巴系增生，以成熟淋巴细胞为主，形态大致正常；巨核系增生明显活跃（图1A），全片可见122个巨核细胞，分类25个巨核细胞，其中幼稚巨核细胞占4％，颗粒巨核细胞占56％，产板巨核细胞占40％，可见巨大多分叶核巨核细胞，血小板簇片状分布易见，报告意见为粒系、巨核系增生明显活跃。骨髓活检：有核细胞增生明显活跃，脂肪组织减少；粒系增生明显活跃，各阶段细胞均可见；红系增生活跃，以晚幼红细胞为主；巨核系增生明显活跃，巨核细胞呈现多形性，少分叶、多分叶、多个核病态巨核细胞及巨核细胞集簇可见（图1B）；淋巴及浆细胞增生受抑；可见少量纤维组织，MF-0级。免疫组化示：幼稚细胞标志物CD34、CD117少量散在阳性；巨核细胞CD61阳性，呈散在或疏松小簇分布（图1C）；背景淋巴细胞：T细胞（CD2、CD3、CD5、CD7、CD4、CD8）散在阳性，B细胞（CD20）少量阳性，NK细胞（CD56）偶见阳性，CD57阴性；Ki-67增殖指数约5％。骨髓流式细胞术示：淋巴细胞占有核细胞7.8％，免疫表型未见明显异常；粒系比例增高，以成熟阶段细胞为主，免疫表型未见明显异常；其他细胞群免疫表型未见明显异常。

**图1:**
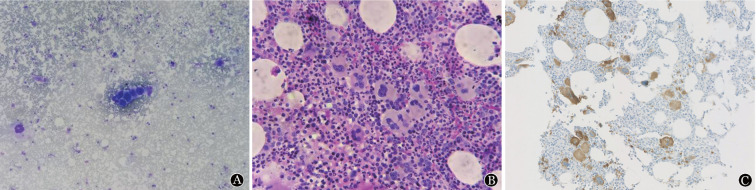
患者骨髓涂片、骨髓活检细胞形态及骨髓组织免疫组化（低倍） **A** 骨髓涂片示粒系增生明显活跃，巨核系增生明显活跃；**B** 骨髓组织活检示有核细胞增生明显活跃，粒系增生明显活跃，巨核细胞呈现多形性，少分叶、多分叶、多个核病态巨核细胞及巨核细胞集簇可见；**C** 骨髓组织免疫组化示巨核细胞CD61胞质性表达，呈散在或疏松小簇分布

通过高通量二代测序（NGS）检测骨髓MPN相关的74种基因，结果提示：SH2B3基因p.S494*突变，为纯合突变，变异等位基因频率（VAF）100％，测序深度为2 272×。SH2B3基因胚系突变验证：口腔脱落细胞NGS阳性位点后续检测，检测到SH2B3基因p.S494*突变。家系遗传分析：通过一代测序位点验证及家系验证，结果显示，患者3位子女SH2B3基因p.S494*突变，为杂合突变，系正常表型。基因检测BCR::ABL p210/p190/p230阴性。结合患者临床表现、实验室检查、骨髓病理、骨髓基因检测，最终诊断为MPN，MPN-10评分35分。

确诊后给予患者芦可替尼单药20 mg每日2次口服，治疗1周后，患者体温恢复正常，乏力缓解，盗汗消失，好转后出院。随访至2025年6月，在近3年的随访中，患者定期复查血常规及腹部B超，结果显示治疗效果良好。血常规各项指标趋于稳定，WBC维持在（5.0～9.5）×10⁹/L，ANC在（3.5～7.0）×10⁹/L。HGB水平基本正常，波动于120～138 g/L。PLT在初始治疗后从543×10⁹/L下降至最低306×10⁹/L，后续虽有波动［（300～600）×10⁹/L］，但总体处于控制状态。同时，脾脏也显著缩小并维持稳定，脾脏厚度从治疗前的6.0 cm减小至4.1 cm，脾长从19.9 cm减小至16.8 cm。尽管患者存在间断停药和减量现象，但临床症状控制良好，体温正常，无乏力、盗汗及腹胀等不适，一般情况良好。

## 讨论及文献复习

MPN是一种克隆性血液系统恶性肿瘤，随着骨髓病理学和分子生物学的广泛研究，其发病机制得以阐释，分类及诊断也逐步细化。在诊断上，2016年WHO已将3个驱动突变JAK2V617F、MPL和CALR纳入了MPN的主要诊断标准，然而一系列研究发现MPN常伴随大量非驱动基因突变，已证实的突变有TET2、DNMT3A、IDH1、IDH2、SH2B3等[3]，其中7％的MPN患者存在SH2B3突变，且多与JAK2V617F、MPL、CALR或MPN中的其他突变并存，而单独发现并致MPN者罕见[4]。该案例系在国内首次发现并证实单独由非驱动基因SH2B3基因纯合胚系突变介导了MPN的发生。

MPN主要为典型驱动基因和（或）非典型驱动基因共同致病，该患者典型驱动基因JAK2、MPL、CALR所有外显子基因突变检测均阴性，亦未发现除SH2B3以外的其他非典型驱动基因的Ⅰ类、Ⅱ类突变及常见变异。患者骨髓SH2B3基因p.S494*突变VAF为100％，口腔脱落细胞SH2B3基因p.S494*突变VAF为99.8％。人类在原胚期，胚胎从囊胚分化为内中外三个胚层，其中造血系统由中胚层发育而来，上皮系统由外胚层发育而来，本例中胚层来源的骨髓细胞与外胚层来源的口腔上皮细胞均检出相同SH2B3基因突变，且突变频率一致，因此可确认该变异为胚系来源，根据AMP指南评级具有强临床意义的突变，此前该变异还未在相关数据库（ClinVar、gnomAD）中被报道。SH2B3基因p.S494*突变表示编码第494号丝氨酸的密码子的核苷酸c.1481C>A发生了单碱基改变，变成了一个终止密码子，蛋白质的合成提前终止，产生非常短且仅含第1到493号氨基酸的截短蛋白，最终导致SH2B3基因编码的蛋白质功能完全丧失。

健康人通过血小板生成素（TPO）和促红细胞生成素（EPO）结合靶细胞表面特异性受体，激活JAK/STAT信号通路维持骨髓细胞的增殖。若JAK2、MPL和CALR突变致JAK/STAT信号通路过度活化是MPN致病的关键分子机制。SH2B3主要表达于造血干细胞、淋系及髓系前体细胞，通过与JAK2相互作用，对TPO和EPO等信号进行负向调控，同时也是造血干细胞中JAK2的生理性负调控因子[5]。SH2B3基因定位于12q24，含8个外显子，编码575个氨基酸，编码的SH2B3蛋白，属于连接蛋白SH2B家族的一员，相对分子质量63×10^3^，结构主要包括：一个富含脯氨酸的N端二聚体结构域、PH（pleckstrin homology）结构域、SH2（Src homology2）结构域、C-末端保守的酪氨酸残基[6]。PH结构域对蛋白质的定位极其重要，SH2结构域负责识别并结合靶蛋白，对维持蛋白功能至关重要[7]。SH2B3本身不具备催化活性，它作为衔接蛋白的主要功能是介导靶蛋白与调控蛋白之间的相互作用，可以通过其SH2结构域直接与JAK2结合。SH2B3能够招募CBL家族E3泛素连接酶，其C3HC4锌结合环指结构域可招募E2泛素结合酶，并催化泛素从E2转移至底物，最终导致靶蛋白泛素化并在蛋白酶体降解[8]，即CBL家族E3泛素连接酶通过衔接蛋白SH2B3下调JAK2信号活性。实验表明，敲除CBL/CBL-B或SH2B3可消除JAK2泛素化修饰，延长其半衰期，并增强JAK2信号传导和细胞增殖能力[9]。SH2B3蛋白功能缺陷可引起EPO、TPO、干细胞因子介导的JAK/STAT3、JAK/STAT5、PI3K/Akt和MAPK信号通路信号增强和持续延长，导致造血祖细胞数量和造血能力显著增加[10]。研究者通过构建SH2B3基因缺陷动物模型，发现SH2B3缺陷小鼠表现出现MPN样表型，表现出白细胞、血小板增多，造血干细胞自我更新能力增强及骨髓、脾脏中巨核、红系祖细胞数量明显增加，而且脾脏中红系细胞集落对EPO敏感性明显增强[11]。此外，JAK/STAT信号通路的持续失控活化亦是驱动病理性炎症和器官损伤的中心环节。

基于SH2B3基因突变与MPN的相关性研究，该患者SH2B3基因纯合胚系突变且具有MPN的临床特征：发热、盗汗、乏力、脾大，ANC和PLT增高，骨髓组织病理考虑MPN，明确诊断为MPN。在MPN的亚型分类上，难点主要集中在是否符合早期PMF（Pre-PMF），结合患者骨髓病理分析：①骨髓活检提示红系增生活跃，而Pre-PMF以红系增生减低为主；②巨核细胞增生明显活跃，呈多型性，少分叶、多分叶、多个核病态巨核细胞可见，但均不占优势，而Pre-PMF以低分叶巨核细胞为主；③巨核细胞集簇以松散集簇为主，而Pre-PMF以密集集簇为主，基于以上考虑排除Pre-PMF，最终分型为MPN-不能分类。

SH2B3基因纯合胚系突变极其罕见，临床特征尚不明确。Perez-Garcia等[12]曾报道一家系，2例先证者携带纯合移码SH2B3变异，幼年时出现肝脾肿大、自身免疫性肝炎、发育迟缓和自身免疫性甲状腺炎。Blombery等[13]报道2例幼年发病非亲缘SH2B3基因纯合移码变异男性先证者，父母均为杂合携带者，患者1表现为脾大、血小板增多、中性粒细胞增多，骨髓活检显示髓系增生伴巨核细胞增生及异型性，合并斑秃、自身免疫性甲状腺功能减退症；患者2表现为脾大、PLT持续升高，骨髓活检显示轻度骨髓增生，伴巨核细胞增生及异型性，合并自身免疫性甲状腺功能减退症、雷诺综合征、自身免疫性肝炎、自身免疫性糖尿病。Leardini等[14]研究了10例SH2B3相关疾病的临床特征，其中8例由胚系双等位SH2B3功能缺失突变引起。患者儿童期出现血小板增多，还表现出多系统的临床特征，包括生长迟缓、不同程度的神经功能障碍和自身免疫性疾病。提示SH2B3基因纯合突变主要表现为骨髓增殖性疾病及多器官的自身免疫综合征。

鉴于JAK/STAT通路过度活化是患者致病的基础，而芦可替尼是一种JAK1/JAK2抑制剂，其核心作用是靶向并抑制JAK/STAT信号通路，同时兼具抗增殖和抗炎的双重作用[15]。本例患者的治疗经验也进一步证实了芦可替尼在SH2B3突变相关MPN中的有效性。

综上，我们在国内诊断了首例SH2B3基因纯合突变驱动MPN，阐述了发病机制和临床表现，目前单药芦可替尼治疗有效。
